# The key residue responsible for the red shift of bioluminescence spectra of light-sensitive Ca^2+^-regulated photoproteins of ctenophores

**DOI:** 10.1038/s41598-025-16796-7

**Published:** 2025-08-23

**Authors:** Ludmila P. Burakova, Eugene S. Vysotski

**Affiliations:** 1 Photobiology Laboratory, Institute of Biophysics of Siberian Branch of the Russian Academy of Sciences, Federal Research Center “Krasnoyarsk Science Center” of Siberian Branch of the Russian Academy of Sciences, Krasnoyarsk, 660036 Russia; 2 Institute of Fundamental Biology and Biotechnology, Siberian Federal University, Krasnoyarsk, 660041 Russia

**Keywords:** Biochemistry, Biophysics

## Abstract

Isoforms of some ctenophore photoproteins show different maxima of bioluminescence spectra— the ones of mnemiopsin and bolinopsin have λ_max_ at 490 and 500 nm, while λ_max_ for velamin isoforms are at 500 and 508 nm. The reasons for the fact have not yet been established. Here we report on the construction and characterization of a set of mutants of berovin from ctenophore *Beroe abyssicola* with a substitution of Ala in position 106, which was selected for mutagenesis based on the comparison of the amino acid sequences of ctenophore photoproteins and the spatial structure model of berovin, to the residues with different properties of their side chains. The appearance of only Ser in this position, and its OH group in particular, is demonstrated to result in a green light emission. Moreover, we propose a plausible mechanism of bioluminescence spectrum shift towards longer wavelengths and the function of certain residues situated near OH group of the 6-(*p*-hydroxy)-phenyl substituent of coelenterazine in this process. We also conclude that the suggested mechanism of emitter formation is valid for other ctenophore photoproteins as well.

## Introduction

**Fig. 1:**
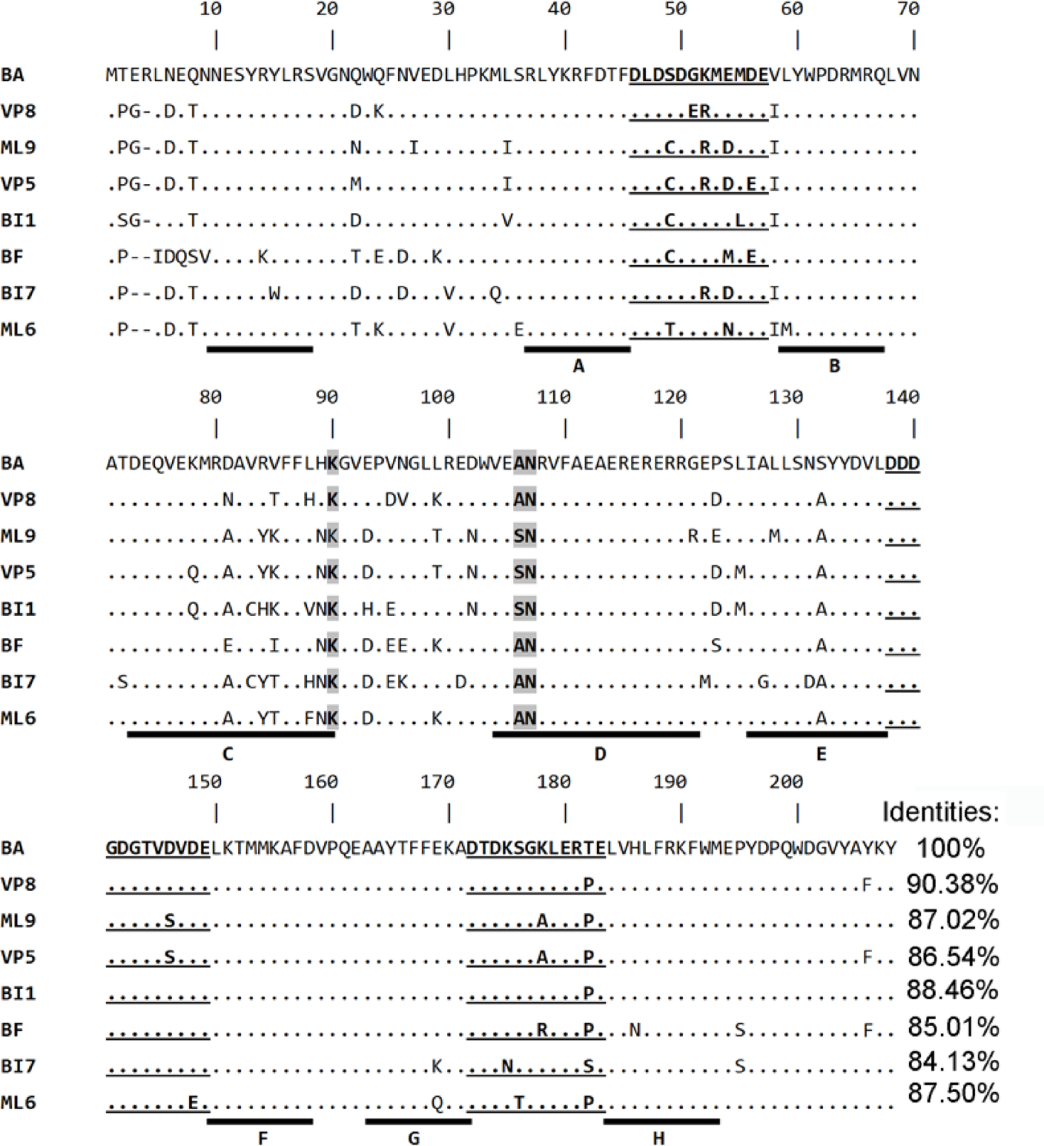
Alignment of amino acid sequences of berovin (BA) (GenBank No. CS050690), mnemiopsin 6 (ML6), mnemiopsin 9 (ML9) (ML6 and ML9 sequences were taken from [42]), bolinopsin (BI7) (GenBank No. CS447621), bolinopsin gr (BI1) (GenBank No. CS447621.1), velamin 5 (VP5), velamin 8 (VP8) (VP5 and VP8 sequences were taken from [8]), and bathocyrovin (BF) (BF sequence was taken from [9]). The alignment was performed using ClustalW. Identical amino acid residues are shown as dots. Ca^2+^-binding sites I, II, and III are bolded and underlined. The residues hypothetically forming an interaction with OH group of the 6-(*p*-hydroxy)-phenyl substituent of coelenterazine oxygenated adduct are bolded and marked in gray. α-helices are shown as black blocks and labeled A-H.

Similar to various luminous jellyfish, the bright bioluminescence of ctenophores is also determined by Ca^2+^-regulated photoproteins^1–3^. Each of the ones, like any hydromedusan photoprotein, is a stable enzyme-substrate complex comprising a protein, within the internal cavity of which an oxygen-activated coelenterazine molecule is tightly but noncovalently bound^3^. Although many ctenophore species are luminous, the only cloned were the cDNAs encoding five photoproteins. These are berovin^4,5, ^bolinopsin^6, ^mnemiopsin^7, ^bathocyrovin^8, ^and velamin^9^ from *Beroe abyssicola*,* Bolinopsis infundibulum*,* Mnemiopsis leidyi*,* Bathocyroe foster*,* and Velamen parallelum*, respectively. Noteworthy is that the first cDNAs encoding berovin and bolinopsin were cloned from the corresponding animals via functional screening of the relevant cDNA libraries in *Escherichia coli* by colony luminescence with coelenterazine^5,10, ^while the other cDNAs encoding ctenophore photoproteins were isolated by PCR with degenerate primers which were designed using the sequences of berovin and bolinopsin. All ctenophore photoproteins cloned to date are one-subunit proteins consisting of 206–208 amino acids with a sequence identity of 84–88% exceeding that of hydromedusan photoproteins (63–70%)^11^ and containing three canonical EF-hand Ca^2+^-binding sites, each with 12 canonical residues^3,5^ (Fig. 1), i.e., similar to hydromedusan photoproteins they belong to a family of the EF-hand Ca^2+^-binding proteins. At the same time, the amino acid sequence identity between ctenophore and hydromedusan photoproteins appeared to be very low (∼29% in the case of obelin from *Obelia longissima*)^5^. This reasonably accounts for some differences in their properties, among which the most important distinctions are a loss of bioluminescence ability on the exposure to light over its entire absorption spectrum and a requirement of alkaline pH to form an active photoprotein from apoprotein and coelenterazine^3^. Although some progress in the studies in this respect was achieved (chemical structure of photoinactivation product was determined^12^ and critical amino acid residue responsible for alkaline pH requirement for conversion of apoprotein into active photoprotein was revealed^13^, the mechanism of these processes is still unclear.

The significant progress in understanding the mechanism of light emission reaction catalyzing hydromedusan photoproteins was achieved owing to determination of their spatial structures. As for now, the crystal structures were determined for active aequorin^14, ^obelin^15,16, ^clytin^17, ^mitrocomin^18 ^and for several conformational states of obelin bound with different ligands^19–21^. Moreover, the spatial structures of several obelin mutants with substitution of key residues were also determined^22–26^. These structural and comprehensive mutagenesis studies allowed identification of the key residues of the internal cavity of hydromedusan photoproteins involved in catalytic decarboxylation of oxygenated coelenterazine and emitter formation^11,26–28^. In particular, the amino acid residues surrounding the OH group of 6-(*p*-hydroxy)-phenyl substituent of 2-hydroperoxycoelenterazine were shown to drastically influence the light emission spectrum and consequently the emitter formation^22–24,29,30^.

In contrast, the spatial structure of active ctenophore photoprotein, i.e. bound with preoxygenated coelenterazine molecule, is not determined yet. The crystal structures of these photoproteins were determined only for apoproteins bound to different ions – apo-berovin with Ca^2 +^^31^ or Mg^2 +^^32^ and apo-mnemiopsin loaded with Cd^2 +^^33^. It is to be noted that the comparison of the spatial structure of apo-berovin with that of aequorin in the same conformational state showed the RMSD value for the main chain atoms to equal 1.77 Å only, despite the low identity of their amino acid sequences^31^. It clearly shows that the residues involved in the formation of internal substrate-binding cavities and thus in stabilization of coelenterazine peroxide adduct, the emitter formation, and the catalysis of light emission reaction in ctenophore and hydromedusan photoproteins are completely different.

Photoproteins from various species may be present either in one or multiple isoforms. This is typical for hydromedusan and ctenophore photoproteins. For instance, only one isoform for obelin from *Obelia longissima*^34,35^ as well as that for obelin from *Obelia geniculata*^36^ was isolated, whereas for aequorin and clytin from *Aequorea victoria* and *Clytia gregaria* the multiple isoforms were cloned and, moreover, they differed not only in amino acid sequences but also in some properties^37–40^. In the case of ctenophore photoproteins, one isoform was cloned for berovin^5^ and bathocyrovin^8, ^while for bolinopsin^41, ^mnemiopsin^42, ^and velamin^9^ the multiple isoforms were also isolated. Whereas most of the ctenophore photoproteins emit blue light with a maximum at 490 nm, several isoforms were identified with red-shifted bioluminescence (λ_max_ = 500 nm) among bolinopsin^41^ and mnemiopsin^42^ isospecies. Noteworthy is that velamin isoforms also have different light emission spectra but, in contrast to those of bolinopsin and mnemiopsin, with λ_max_ at 500 and 508 nm^9^. The comparison of amino acid sequences of these isoforms revealed that among the existing variations the one amino acid position may be responsible for the shift of light emission spectrum towards longer wavelengths. This amino acid is in position 106 (according to berovin sequence) which is occupied by Ala in the “blue” isoforms, while Ser is found in this position in “green” isospecies (Fig. 1). Although velamin isoforms already emit green light, the bioluminescence spectrum of isoform in which this position is occupied by Ala is blue-shifted (λ_max_ = 500 nm) as compared to that of isoform in which Ser is found in this position (λ_max_ = 508 nm). According to one of the three-dimensional models of active berovin^13 ^the residue in position 106 is situated in close proximity to OH group of the 6-(*p*-hydroxy)-phenyl substituent of coelenterazine oxygenated adduct and consequently in the case of Ser its side chain comprising the OH group in contrast to Ala may form additional hydrogen bond either with OH group of the 6-(*p*-hydroxy)-phenyl substituent or some residue involved in emitter formation.

To find out if the residue in position 106 is really responsible for the difference in bioluminescence spectra of ctenophore photoprotein isoforms, we constructed a set of berovin mutants (A106S, A106H, A106R, A106W, A106Y, A106D, A106V, A106T, A106C, and A106N) with substitution of Ala106 to residues with different properties of side chains and investigated the effect of these replacements on bioluminescence.

## Methods

### Materials

Oligonucleotides were purchased from CCU “Genomika” (Novosibirsk, Russia). Coelenterazine (CTZ) was obtained from NanoLight Technology, a division of Prolume Ltd. (Pinetop, AZ, USA). Other chemicals of the purest grade available were from Sigma-Aldrich, unless otherwise stated.

### Molecular biology

Site-directed mutagenesis was carried out on the pET22b-BA plasmid^5^ for *Escherichia coli* expression carrying the *B. abyssicola* wild-type apo-berovin gene. Mutations resulting in the desired amino acid change were carried out using specific primers with the QuikChange site-directed mutagenesis kit (Agilent Technologies, USA) according to the protocol supplied with the kit. The resulting plasmids were verified by DNA sequencing (SB RAS Genomics Core Facility, Novosibirsk, Russia).

### Protein expression and purification

For apophotoprotein production, the transformed *E. coli* BL21 (DE3) Codon Plus (RIPL) cells were cultivated with vigorous shaking at 37 °C in LB medium containing ampicillin (200 µg/mL). Protein expression was induced with 1 mM IPTG at OD_600_ of 0.6–0.8 and the cultivation was continued for another 3 h. Apoproteins were purified from inclusion bodies as previously described for the recombinant wild-type berovin^5,43^. The *E. coli* cells were harvested by centrifugation, resuspended in 20 mM Tris-HCl pH 7.2, and disrupted by ultrasound (20 s × 6) on ice. The suspension was centrifuged (10,000 g × 10 min) at 4 °C and supernatant was discarded. The pellet containing inclusion bodies was sequentially washed by centrifugation (10,000 g × 10 min) at 4 °C with (1) 0.9% NaCl, Triton X-100, 20 mM Tris-HCl pH 7.2 and (2) 20 mM Tris-HCl pH 7.2. To extract apophotoprotein, the pellet was resuspended in 6 M urea, 20 mM Tris-HCl pH 7.2 for 30 min (approximately 1 mL of solution per inclusion bodies obtained from 1 g of *E. coli* cells). Then suspension was centrifuged (10,000 g × 10 min) at 4 °C, the pellet was discarded, and supernatant was used for further chromatographic purification of apoprotein. Noteworthy is that after washing procedure apophotoprotein contains only minor impurities. To produce apophotoprotein of high purity, the ion-exchange chromatography in 6 M urea on HiTrap DEAE Fast Flow 5 mL column (GE Healthcare, USA) was applied. The obtained supernatant was loaded onto the column equilibrated with 6 M urea, 20 mM Tris-HCl pH7.2 and eluted by linear gradient of 0.8 M NaCl with 6 M urea, 20 mM Tris-HCl pH7.2. The apophotoproteins are eluted between 0.25 and 0.5 M NaCl. After ion-exchange chromatography the peak containing apophotoprotein was collected and concentrated up to approximately 1 mL by centrifugation at 5,000 g using Amicon Ultra 15 mL Centrifugal Filters (Merck Millipore, USA) at 4 °C. The concentrated apoprotein sample was immediately added to the buffer 0.5 M NaCl, 5 mM EDTA, 50 mM Tris-HCl pH 9.0 (1:10 v/v) with coelenterazine (molar ratio apophotoprotein/coelenterazine is ~ 1:1.1), and quickly mixed. Coelenterazine concentration in the methanol stock solution was determined spectrophotometrically (ε_435_ nm = 9800 M^− 1^ cm^− 1^)^1]^. The wild-type berovin and each of its mutants were activated by coelenterazine for 24 h and then purified. The active photoprotein was separated from apophotoprotein and coelenterazine excess via ion-exchange chromatography by applying a linear gradient of NaCl from 0 M to 0.5 M on a Capto HiRes Q 5.50 column (GE Healthcare, USA) with a flow rate of 1 mL/min. The elution buffers were: A – 1 mM EDTA, 20 mM Tris-HCl pH 7.2; and B – 1 M NaCl, 1 mM EDTA, 20 mM Tris-HCl pH 7.2. Freshly purified proteins were immediately used in the experiments. The active photoprotein yield was estimated using the following equation:

1 $$Y(\% ) = \frac{{C_{{active}} }}{{C_{{active}} + C_{{apoprotein}} }} \times 100\%$$

where *C*_active_ and *C*_apoprotein_ are protein concentrations in the fraction corresponding to the active photoprotein and apophotoprotein obtained by chromatography on a Capto HiRes Q 5.50 column. The protein concentration was determined with the Dc Bio-Rad protein assay kit (Bio-Rad, USA). All procedures with active wild-type berovin and its mutants including bioluminescence and spectral measurements were performed in the dark or under dim red light to avoid photoinactivation.

### Bioluminescence assay

Bioluminescence was measured with a luminometer (BLM-8812, Krasnoyarsk, Russia) by rapid injection of 10 µL of photoprotein solution in 1 mM EDTA, 20 mM Tris-HCl pH 7.2 into a luminometer cell containing 490 µL of 2 mM CaCl_2_ in 50 mM Tris-HCl pH 8.5 at room temperature. The light signal was recorded until it completely ceased. Specific bioluminescence activity (*L*_specific_) was estimated as a ratio of total light determined by integrating bioluminescent signal to protein concentration in a sample by averaging three independent measurements. The Ca^2+^-independent luminescence (*L*_Ca−free_)^44^ was calculated as a ratio of maximal light signal from 500 µL of photoprotein in 0.3 M NaCl, 1 mM EDTA, 20 mM Tris-HCl pH 7.2 normalized to protein concentration to the specific bioluminescence activity^36^.

### Spectral measurements

Bioluminescence and fluorescence spectra were recorded with a Varian Cary Eclipse spectrofluorometer (Agilent Technologies, USA). The slit width was 5 nm. To record the bioluminescence spectra, the 50–100 µL purified protein in ~ 0.3 M NaCl, 1 mM EDTA, 20 mM Tris-HCl pH 7.2 was placed into spectrofluorometer cell containing 950 − 900 µL of 50 mM bis-Trispropane pH 8.5. Bioluminescence was initiated by injection of 20 µL of 100 mM CaCl_2_ solution into the same buffer. The light emission spectra were recorded in the range of 370–600 nm with a rate of 12,000 nm/min. The bioluminescence spectra were recorded at an approximately constant light level during the spectral scan. In cases when a substantial change in bioluminescence intensity took place at spectral measurements, the data points were also corrected for bioluminescence decay. The fluorescence spectra of Ca^2+^-discharged photoproteins were recorded after the bioluminescence reaction ceased. The measurements were performed in the range of 370–600 nm with a rate of 1200 nm/min. The bioluminescence and fluorescence spectra were corrected for spectral sensitivity of the detector using an algorithm supplied with the instrument.

### Photo- and thermoinactivation

Photoinactivation was performed for 1 h at 0 °C (on ice) with an incandescent lamp. The 200 µL protein solution with a final concentration of 0.1 mg/mL in ~ 30 mM NaCl, 1 mM EDTA, 20 mM Tris-HCl pH 7.2 was placed into a 0.5 mL Eppendorf type tube about 10 cm away from the light source. Thermal inactivation was performed for 1 h at 37 °C. The 200 µL protein solution with a final concentration of 0.1 mg/mL in ~ 30 mM NaCl, 1 mM EDTA, 20 mM Tris-HCl pH 7.2 was placed into a 0.5 mL Eppendorf type tube in a dry-air thermostat (Binder KB53, Germany). In both cases bioluminescence was measured every 10 min as described above.

## Results

**Fig. 2:**
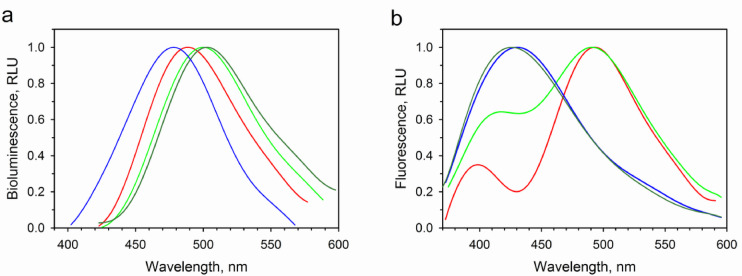
Bioluminescence and fluorescence spectra of BAwt and certain mutants. (**a**) Normalized bioluminescence spectra and (**b**) fluorescence spectra of Ca^2+^-discharged proteins of BAwt (red), A106S (green), A106T (dark green), and A106V (blue) mutants. The excitation wavelength is 350 nm.

**Fig. 3:**
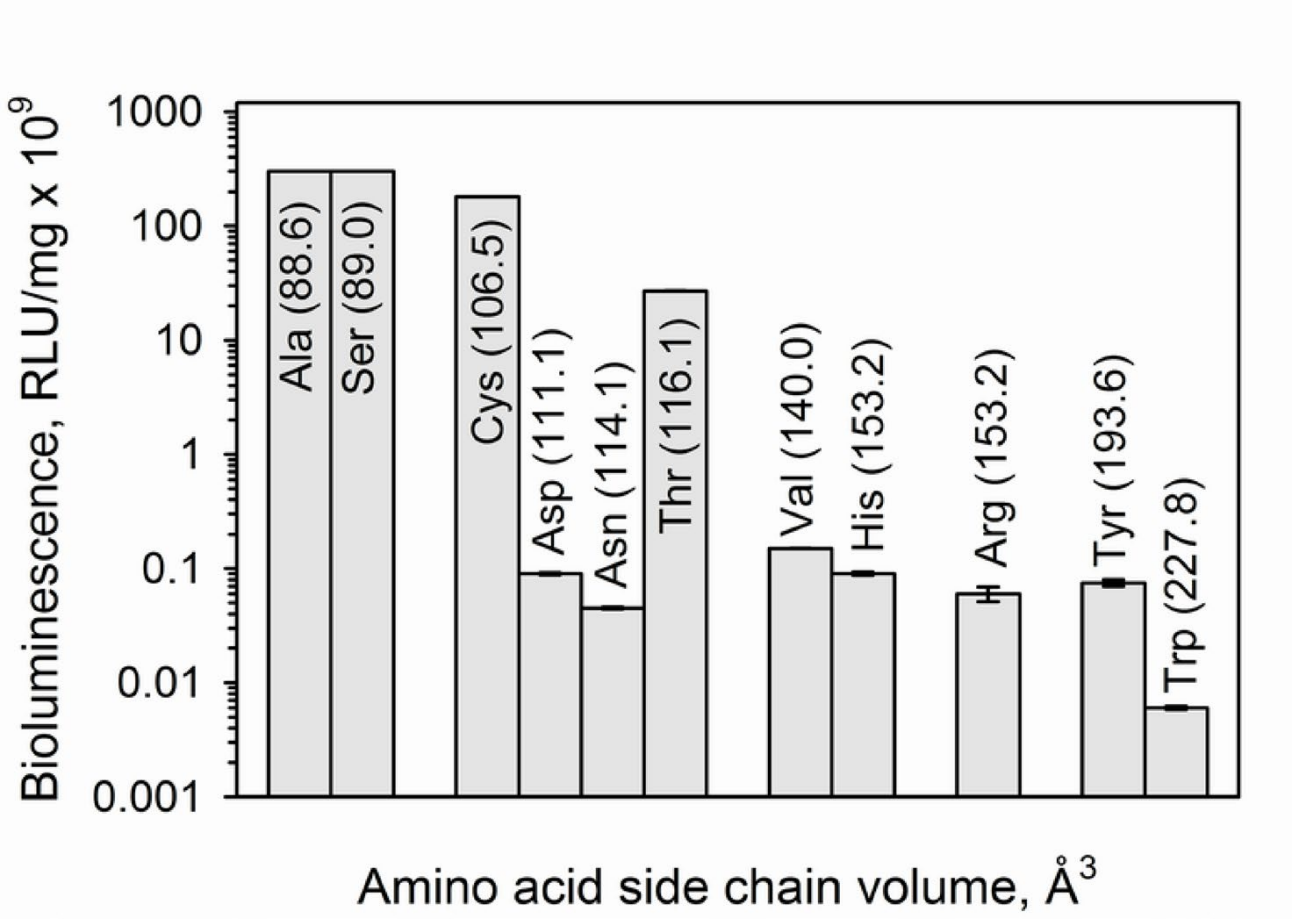
Semi-logarithmic plot for Ala106 substitution effect on berovin bioluminescence activity depending on amino acid side chain volume. The bars show the amino acid residues at position 106 in berovin in increasing order of side chain volume.

As was hypothesized from the analysis of amino acid sequences (Fig. 1) and spatial berovin structure model^13 ^the replacement of Ala106 in berovin for Ser actually shifts its light emission spectrum towards longer wavelengths (λ_max_ = 500 nm) (Table 1; Fig. 2), thereby making bioluminescence spectrum of berovin identical to those of the “green” isoforms of bolinopsin^41^ and mnemiopsin^42^. The main peak of fluorescence spectrum of Ca^2+^-discharged A106S mutant coincides with that of Ca^2+^-discharged berovin of the wild-type (Fig. 2), i.e., in contrast to the wild-type berovin, the spectral fluorescence peak is shifted towards shorter wavelengths as compared to the bioluminescence one. However, both Ca^2+^-discharged photoproteins, A106S mutant and the wild-type berovin, display a shoulder at 400 nm in the fluorescence spectrum (Fig. 2) corresponding to the emission of neutral coelenteramide^1^. Thus, this substitution does not affect the specific activity and the yield of active photoprotein but enhances the intensity of Ca^2+^-independent luminescence 4.5 times as compared to the wild-type berovin (Table 1). It should be noted that the volume of Ser side chain is identical to that of Ala (Fig. 3).

The appearance of Thr which side chain, like in Ser, also includes the OH group, but additionally has the CH_3_ group and consequently higher side chain volume (Fig. 3) in position 106 instead of Ala, makes the light emission spectrum of berovin shift towards longer wavelengths even to a greater extent (λ_max_ = 505 nm) as compared to that in the case of A106S mutant (Table 1). At the same time, this substitution significantly reduces the yield of active protein and specific activity of the mutant photoprotein as well as noticeably increases the level of its Ca^2+^-independent luminescence. It is worth mentioning that the fluorescence spectrum of Ca^2+^-discharged A106T mutant strikingly differs from those of the wild-type berovin and A106S mutant. Whereas Ca^2+^-discharged A106T mutant displays maximum at 425 nm, the fluorescence maxima of the wild-type berovin and A106S mutant are found within the 490–497 nm spectral range with a shoulder at 400 nm (Table 1).

**Table 1:** Properties of berovin mutants.

Photoprotein	Yield of active protein, %*	L_specific_, RLU/mg, ×10^9^	L_Ca-free_, ×10^− 8^	BL λ_max_, nm	FL*** λ_max_/shoulder λ_max_, nm (pH 8.5)
BAwt	65	300 ± 0.22 (100%)	0.4	490	398/**492**
A106S	60	300 ± 0.23 (100%)	1.8	500	400/**492**
A106T	15	27.0 ± 0.08 (9%)	25	505	**425**
A106C	60	180 ± 0.22 (60%)	42	488	420/**485**
A106V	7	0.15 ± 0.001 (0.05%)	125	480	**430**
A106D	5	0.09 ± 0.002 (0.03%)	160	ND**	**410**
A106N	13	0.045 ± 0.001 (0.015%)	5.5	485	**418**
A106H	7	0.09 ± 0.003 (0.03%)	66	ND	**425**
A106R	17	0.06 ± 0.009 (0.02%)	4.1	480	**420**
A106Y	30	0.075 ± 0.005 (0.025%)	60	490	**418/**500
A106W	28	0.006 ± 0.0002 (0.002%)	48	492	**395**/520

*The yield was estimated by averaging of at least two independent protein preparations.

**ND, not detected.

***The main fluorescence maximum is highlighted in bold.

The replacement of Ala by Val, the side chain of which is similar to that of Ala in property but differs in size (Fig. 3), leads to respectively 10-fold and 2,000-fold drop of the active photoprotein yield and specific activity versus the wild-type berovin. At the same time, the Ca^2+^-independent luminescence raises more than 300-fold (Table 1). In addition, this substitution shifts the bioluminescence spectrum towards shorter wavelengths (λ_max_ = 480 nm) and alters the fluorescence spectrum of Ca^2+^-discharged A106V mutant (λ_max_ = 430 nm).

The substitution of Ala106 to Asp which side chain volume is ∼1.25-fold higher than that of Ser (Fig. 3) and differs in donor-acceptor properties sharply reduces the specific activity and the active photoprotein yield. Besides this, the A106D mutant showed a high level of Ca^2+^-independent luminescence exceeding that of the wild-type berovin 4,000-fold (Table 1), thereby pointing to a significant distortion of stability of photoprotein complex. The light emission spectrum of this mutant was not determined owing to its low specific activity and the yield of active photoprotein. Nevertheless, the Ca^2+^-discharged A106D mutant demonstrated bright fluorescence with λ_max_ at 420 nm (Table 1), i.e., most likely, the substitution of Ala to Asp has no effect on coelenterazine binding within the internal cavity and its activation by oxygen, but influences the stability of peroxy adduct of coelenterazine within the active site.

The replacement of Ala106 by Asn with the side chain volume almost equal to that of Asp (Fig. 3), but with different donor-acceptor properties, also resulted in a 5-fold decrease in the active protein yield and an almost a 6,700-fold decrease in activity compared to the wild-type berovin. At the same time, the level of Ca^2+^-free bioluminescence in this mutant is almost 30 times lower than in A106D. The maximum of bioluminescence spectrum is shifted to the blue region by 5 nm compared to the wild-type berovin, and the fluorescence spectrum is similar to that of A106D.

The Cys in place of Ala ensures the same yield of active photoprotein as the wild-type berovin does, however its specific activity is 1.7 times lower than that of the wild-type berovin, and Ca^2+^-independent luminescence is two orders of magnitude higher. At the same time, the fluorescence of Ca^2+^-discharged protein is extremely low after bioluminescence reaction that may be indicative of rapid dissociation of coelenteramide from the active center. In other respects, this mutant differs little from the wild-type berovin (Table 1) that may be conditioned by small distinctions in side chain volumes of Cys, Ala, and Ser (Fig. 3).

All berovin mutants with the replacement of Ala106 by Arg, Trp, Tyr, or His residue, in which the volume of side chains as well as their donor-acceptor properties significantly differ from that of Ala, exhibited reduced specific activities and active photoprotein yields (Table 1; Fig. 3). The most significant decrease of specific activity among them was observed for A106Y and A106W mutants which activities dropped respectively by 4,000 and 50,000 times as compared to the wild-type berovin. At the same time, the yields of active photoproteins were only approximately twice lower (Table 1). The substitution of Ala106 to Tyr or Trp does not change the spectra of light emission but drastically influences the fluorescence spectra of Ca^2+^-discharged mutants (Table 1). The A106Y displays a main peak at 418 nm with a small shoulder at 500 nm, while A106W has a maximum at 395 nm and a big red-shifted shoulder (λ_max_ = 520 nm). The replacement of this Ala by Arg also decreases specific activity of A106R mutant but to a lesser extent than in the case of Ala substitution to Trp or Tyr (Table 1); the activity drops only by an order of magnitude as compared to the wild-type berovin. Meanwhile, the yield of active A106R mutant is slightly lower than those for A106Y and A106W mutants. The A106R mutant’s bioluminescence spectrum is similar to the one of the wild-type berovin whereas the fluorescence spectrum has a main peak at 420 nm which practically corresponds to the main fluorescence peak of the Ca^2+^-discharged A106N mutant (Table 1). Appearance of His instead of Ala in position 106 leads to the decrease of the active photoprotein yield and specific activity; the values of these parameters were approximately the same as in the case of A106D mutant. The low activity and the yield of active photoprotein did not allow us to measure the light emission spectrum of A106H mutant. The Ca^2+^-discharged A106H mutant at that showed bright fluorescence with λ_max_ at 425 nm (Table 1), i.e., the appearance of His does not influence coelenterazine binding and its activation by oxygen but affects the stability of oxygenated adduct of coelenterazine.

**Fig. 4:**
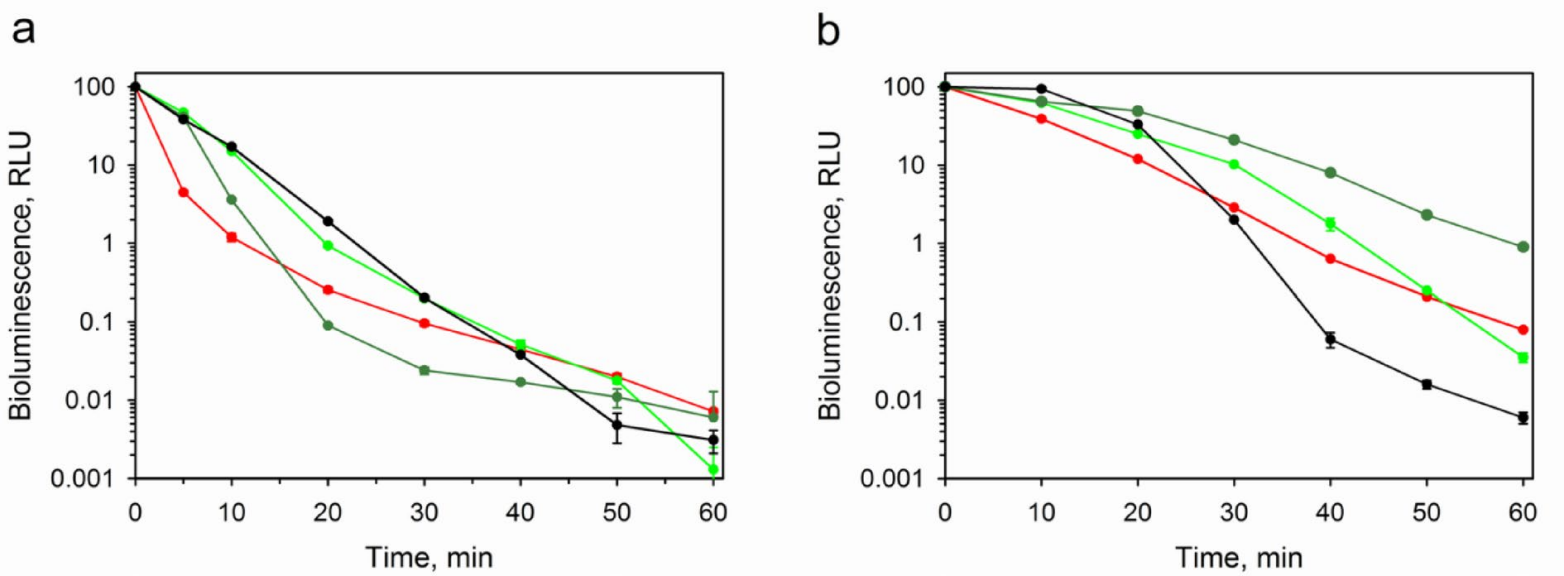
Semi-logarithmic plot of normalized bioluminescence activity decay of wild-type berovin (red), A106S (green), A106T (dark green), and A106C (black) mutants under light irradiation (**a**) and during incubation at 37 °C (**b**).

Since the distinctive feature of ctenophore photoproteins is inactivation of active protein at exposure to light over its entire absorption spectrum^1 ^we also compared the wild-type berovin and its A106S, A106T, and A106C mutants which revealed the highest specific activities (Table 1) in terms of their resistance to light as well as to thermal inactivation (Fig. 4). Although the wild-type berovin and its mutants practically lost the activity in 1 h of irradiation (residual activities are in the range of 0.007 − 0.001%), the kinetics as well as the sensitivity to photoinactivation estimated as a time during which photoprotein loses 50% of bioluminescent activity appeared to be different (Fig. 4a). The wild-type berovin turned out to be most sensitive to photoinactivation (t_50%_ = ∼2.6 min), whereas its A106S mutant displayed the highest resistance to light irradiation (t_50%_ = ∼4.7 min). The sustainability to light of other mutants also exceeded that of the wild-type berovin (t_50%_ = ∼4.2 and 4.0 min for A106T and A106C mutants, respectively). The effect of mutations on thermal inactivation (Fig. 4b) was assessed as in the case of photoinactivation. The wild-type berovin loses 50% of activity during ∼8 min while its A106S, A106T, and A106C mutants retain 50% of activities in approximately 13, 19, and 17 min, respectively. These distinctions, as in the case of photoinactivation, disappear in 1 h of thermal inactivation; the residual activities are very low varying in the range of 1.5-0.006%. The reason of these distinctions is unclear but may be conditioned, for example, by structural changes of photoprotein molecules as a result of introducing of other residues instead of Ala106.

## Discussion

The imidazopyrazinone-type luciferins such as coelenterazine, for example, were found in various taxonomically distant luminous marine organisms as substrates of light-emitting reactions catalyzed by both luciferase and photoproteins^1,2,45^. Despite these bioluminescent proteins have no any amino acid and structural homology, it now is generally agreed that the reaction chemical mechanism giving the product in an excited state is the same for the enzymes utilizing substrates of this type. The chemical mechanism derives from the chemiluminescence studies of coelenterazine analogues in aprotic solvents in the presence of O_2_ and a trace amount of a base^46,47^. The reaction is initiated by deprotonation of N7 leading to the formation of a C2 anion of coelenterazine. Then, single electron transfer (SET) from C2 anion to O_2_ produces coelenterazine radical and superoxide anion radical of oxygen, which fast coupling affords the peroxide anion. In the case of chemiluminescence or bioluminescence reactions catalyzed by luciferases the peroxide anion cyclizes yielding dioxetanone, which instantly decomposes with the release of CO_2_ and an amide product in the excited state. In the case of Ca^2+^-regulated photoproteins in the absence of calcium ions the reaction stops after peroxide anion formation due to interactions with the side chains of the active site amino acid residues^1,27,48^. Binding of Ca^2+^ to protein destabilizes the peroxide anion within the active site that results in dioxetanone formation and its further decomposition with generation of the product in an excited state.

The most significant progress in understanding the function of amino acid residues located in the substrate-binding cavity in catalyzing bioluminescence reaction and emitter formation was attained for hydromedusan Ca^2+^-regulated photoproteins through comprehensive structural and mutagenesis studies. It allowed proposing the proton-relay mechanism for triggering the bioluminescence reaction by Ca^2+^ and the formation of different excited states of a product^27,28^. As was proposed, the primary excited product in bioluminescence of hydromedusan photoproteins is coelenteramide in a neutral state emitting light with λ_max_ at 390–420 nm. The light emission at longer wavelengths occurs from the excited phenolate anion that arises from transient proton dissociation of the OH group of the 5-(*p*-hydroxy)-phenyl substituent of coelenteramide in the direction to His found within hydrogen bond distance in all hydromedusan photoproteins. Since the p*K* of a phenolic group in excited state is several units below its ground state p*K*^48^ and if the pK* value falls below that of His p*K*, rapid transient proton dissociation and its “transient displacement” towards the N atom of His may take place simultaneously accompanied by generation of the excited phenolate anion. As its fluorescence lifetime is 5–6 ns^49 ^there is more than enough time for proton dissociation before radiation. Along with His, the OH group of the 6-(*p*-hydroxy)-phenyl substituent of coelenterazine is also surrounded by Trp and Tyr in aequorin and mitrocomin or Trp and Phe in obelin and clytin which side chains are found at the hydrogen bond distances. Actually, the substitution of Trp to Phe in aequorin and obelin leads to appearance of bimodal bioluminescence spectrum with λ_max_ at 410 and 470 nm for W92F obelin and 400 and 465 nm for W86F aequorin^11^. The appearance of violet band in bioluminescence spectrum characteristic of light emission from the neutral state of the emitter suggests that this substitution may hamper dissociation of the proton from the OH group of the 6-(*p*-hydroxy)-phenyl substituent.

**Fig. 5:**
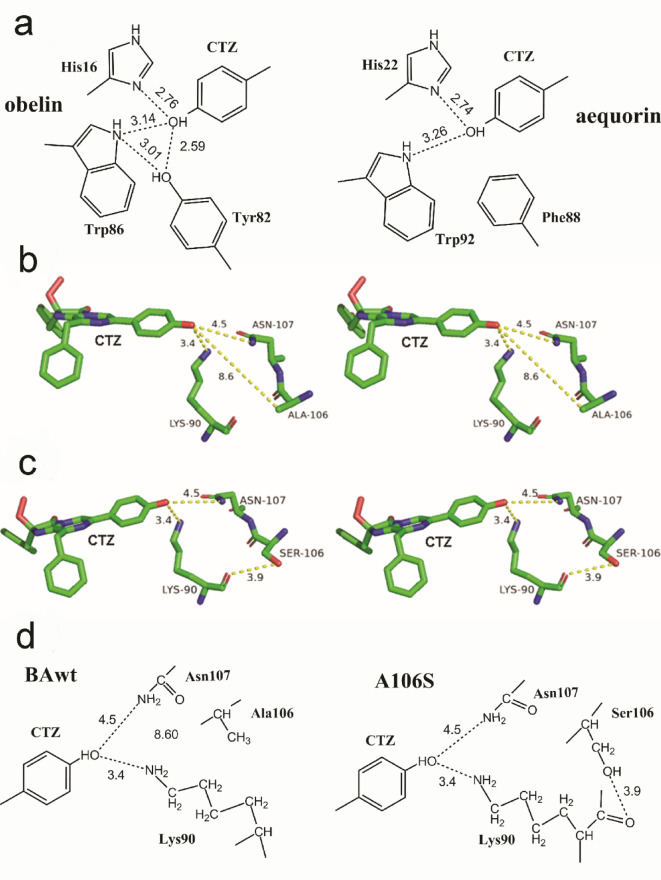
The amino acid residues involved in emitter formation in hydromedusan and ctenophore photoproteins. (**a**) Two-dimensional drawing of the amino acid environment of OH group of the 6-(*p*-hydroxy)-phenyl substituent of coelenterazine in obelin (PDB code 1QV0) and aequorin (PDB code 1EJ3); (**b**, **c**) Stereoview of the residues surrounding OH group of the 6-(*p*-hydroxy)-phenyl substituent of coelenterazine correspondingly in the wild-type berovin (**b**) and its A106S mutant (**c**). Stereoviews were generated in PyMOL 2.5.0 (https://www.pymol.org/ ) (Schrodinger, LLC)  using berovin structural model^13^ as a template ; (**d**) Two-dimensional drawing of the amino acid environment of OH group of the 6-(*p*-hydroxy)-phenyl substituent of coelenterazine in the wild-type berovin and its A106S mutant. Distances are in Å.

Close environment of OH group of the 6-(*p*-hydroxy)-phenyl substituent of coelenterazine in aequorin and obelin differs in one residue—the 88 position in obelin is occupied by Phe whereas Tyr is found in the corresponding position in aequorin (Fig. 5a). Whereas aequorin has a maximum of light emission spectrum with λ_max_ at 470 nm, obelin displays the 10-nm red-shifted spectrum (λ_max_ = 480 nm) with a shoulder at 390 nm^50^. The substitution of Tyr to Phe shifts the light emission spectrum of aequorin towards longer wavelengths making it very similar to that of obelin^30^. In contrast, the obelin mutant with replacement of Phe by Tyr displays bioluminescence spectrum which maximum is very close to that of aequorin and has no shoulder. This undoubtedly shows that the difference in light emission spectra of these photoproteins is the result of an additional hydrogen bond between the hydroxyl group of Tyr and the oxygen atom of the 6-(*p*-hydroxy)-phenyl group of coelenterazine. Moreover, the spatial structures of F88Y obelin determined for conformational states before and after the bioluminescence reaction confirm this conclusion^24^. Of note is that according to the QM/MM (quantum mechanics/molecular mechanics) calculations the light emission maximum of coelenteramide fluorescence strongly depends on the distance at which the proton is located relative to the oxygen of the 5-(*p*-hydroxy)-phenyl substituent^51^. Hence, we may reasonably draw a conclusion that the different arrangement of the hydrogen bond network accounts for different position of the proton relative to the N atom of His and the oxygen of the 5-(*p*-hydroxy)-phenyl group of excited coelenteramide, and therefore for different light emission colors.

The mechanism of emitter formation in ctenophore photoprotein bioluminescence may be the same as in hydromedusan photoproteins but involving different amino acids. According to the structural model of berovin^13 ^the 6-(*p*-hydroxy)-phenyl substituent is surrounded by Lys90 and Asn107 which side chains are found in close proximity to the oxygen atom of OH group (Fig. 5b). Since only the overall structure of proteins but not the positions of certain side chains of amino acids can be well predicted by AlphaFold, we can plausibly assume the N atoms of side chains of Lys90 and Asn107 to be located at shorter distances to the oxygen atom of OH group of the 6-(*p*-hydroxy)-phenyl substituent and, consequently, to perform the function similar to that of the residues surrounding the OH group in hydromedusan photoproteins, i.e., to promote the transient proton dissociation with the formation of excited phenolate anion of coelenteramide.

According to the modeling, the replacement of Ala106 to Ser changes orientation of the residue side chain in this position. Whereas the Ala hydrophobic side chain is directed into the internal cavity, the Ser OH group is oriented oppositely (Fig. 5c). Moreover, it may form the hydrogen bond with the main chain oxygen atom of Lys90 (Fig. 5d), thereby binding α-helices C and D (Fig. 1). The additional hydrogen bond may affect the position of Lys side chain and, consequently, the distance to which a proton may dissociate in direction of its NH_2_ group that, in turn, may change the light emission color^51^. Also worth mentioning is that the OH group of Thr in A106T mutant may be hydrogen-bonded with the main chain oxygen atom of Lys90 as well. Noteworthy is that the mechanism leading to the red shift of light emission maximum of velamin isoforms may be the same as proposed for other ctenophore photoproteins. For instance, the green bioluminescence observed even in the presence of Ala in this position may be caused by either different distance of Lys side chain relative to OH group of the 6-(*p*-hydroxy)-phenyl substituent due to a little difference in protein conformation or the influence of some other active site residues in this photoprotein on emitter formation.

## Conclusion

Our study undoubtedly shows that the presence of Ser and its OH group in position 106 is responsible for the green light emission of Ca^2+^-regulated photoproteins from ctenophores. The critical importance of OH group for a shift of light emission spectrum towards longer wavelengths is supported by substitution of Ala106 to Thr; the light emission spectrum of A106T mutant is shifted even a little more than at substitution Ala to Ser (Table 1; Fig. 2). Furthermore, by applying site-directed mutagenesis we also demonstrate that the side chain volume of amino acid in this position is critical for photoprotein activity—only substitution of Ala106 to Ser which side chain volume corresponds to that of Ala does not affect the specific activity (Table 1; Fig. 3). Basing on the structural model of berovin^13 ^we also discuss of how the OH group may affect the emitter formation in bioluminescence reaction of ctenophore photoproteins. The proposed mechanism looks reasonable since in general it corresponds to the one suggested for hydromedusan photoproteins which is based on comprehensive mutagenesis and structural studies^11,24,27,28^ with only one distinction—the residues, Asn and Lys, involved in the emitter formation differ from those in hydromedusan photoproteins.

It should be emphasized that although the proposed mechanism well accounts for the effect of OH group of Ser on the bioluminescence spectrum of ctenophore photoprotein, it is only the determination of spatial structure of ctenophore photoprotein with a bound substrate or the reaction product that would ensure satisfactory evidence for the role of amino acid residues surrounding OH group of the 6-(*p*-hydroxy)-phenyl substituent in the emitter formation in these photoproteins.

## Data Availability

The datasets generated and analyzed during this study are included within the article and are also available from the corresponding author upon reasonable request.
